# Patient-Reported Outcomes and Complications Following Breast Reconstruction: A Comparison Between Biological Matrix-Assisted Direct-to-Implant and Latissimus Dorsi Flap

**DOI:** 10.3389/fonc.2022.766076

**Published:** 2022-01-27

**Authors:** Peng Gao, Ping Bai, Xiangyi Kong, Yi Fang, Jidong Gao, Jing Wang

**Affiliations:** ^1^ Department of Breast Surgical Oncology, National Cancer Center/National Clinical Research Center for Cancer/Cancer Hospital, Chinese Academy of Medical Sciences and Peking Union Medical College, Beijing, China; ^2^ Department of The Operation Room, National Cancer Center/National Clinical Research Center for Cancer/Cancer Hospital, Chinese Academy of Medical Sciences and Peking Union Medical College, Beijing, China

**Keywords:** breast cancer, direct-to-implant breast reconstruction, latissimus dorsi flap, biological matrix, BREAST-Q version 2.0

## Abstract

**Background:**

Implant-based breast reconstruction is increasingly becoming the most common method of postmastectomy breast reconstruction in use today. As the traditional autologous reconstruction technique, latissimus dorsi flap (LDF) is employed by surgeons for reconstruction after breast cancer surgery, including partial mastectomy, modified radical mastectomy, and others. The authors aim to compare patient-reported outcomes (PROs) and complications between the SIS matrix-assisted direct-to-implant (DTI) breast reconstruction and the autologous LDF breast reconstruction.

**Methods:**

Patients undergoing the SIS matrix-assisted DTI reconstruction or mastectomy with LDF reconstruction or partial mastectomy with mini latissimus dorsi flap (MLDF) reconstruction were enrolled in a single institution from August 2010 to April 2019. Patients were included for analysis and divided into three groups: those who underwent LDF reconstruction, those who underwent MLDF reconstruction, and patients who underwent SIS matrix-assisted DTI breast reconstruction. PROs (using the BREAST-Q version 2.0 questionnaire) and complications were evaluated.

**Results:**

A total of 135 patients met the inclusion criteria: 79 patients (58.5%) underwent SIS matrix-assisted DTI, 29 patients (21.5%) underwent LDF breast reconstruction, and 27 patients (20%) underwent MLDF breast reconstruction. PROs and complication rates between LDF reconstruction group and MLDF reconstruction group showed no statistically significant differences. Furthermore, BREAST-Q responses found that patients in the whole autologous LDF reconstruction group had better psychosocial well-being, showing a mean score of 84.31 ± 17.28 compared with SIS matrix-assisted DTI reconstruction, with a mean score of 73.52 ± 19.96 (p = 0.005), and expressed higher sexual well-being (69.65 ± 24.64 vs. 50.95 ± 26.47; p = 0.016). But there were no statistically significant differences between the two groups for postoperative complications.

**Conclusion:**

This retrospective study showed no statistically significant differences between LDF breast reconstruction and MLDF breast reconstruction. However, patients in the whole autologous LDF reconstruction group yielded superior PROs than patients in the SIS matrix-assisted DTI reconstruction group in the psychosocial well-being and sexual well-being domains.

## 1 Introduction

Reconstruction after mastectomy is increasingly becoming the mainstream approach for younger women who are diagnosed with breast cancer. Mastectomy defects can cause significant psychological trauma, making breast reconstruction become the most valuable and challenging procedure performed by breast oncology surgeons in our institute. The increasingly significant demand of Chinese patients for breast reconstruction has promoted the development of a myriad of acceptable procedures.

For the last 5 decades, a variety of techniques for breast reconstruction have been developed to lessen the negative influence of mastectomy on patients’ quality of life (QOL), broadly classified as implant-based reconstructions or autologous tissue reconstructions (1, 2). For patients, the therapeutic course following breast cancer and mastectomy affects their QOL in terms of psychosocial, physical, aesthetic, body image, and sexual issues (3, 4). For surgeons, they need to evaluate different reconstruction approaches according to the characteristics of patients and to assess the time and economic costs for patients. Latissimus dorsi flap (LDF) breast reconstruction, as one of the autologous tissue reconstructions, is the first line for patients who are not candidates for the transverse rectus abdominis muscle (TRAM) flap, due to previous abdominoplasty, prior TRAM, insufficient abdominal skin or fat, and high-risk comorbidities such as diabetes, obesity, or tobacco use (5). The LDF reconstruction offers a reliable and aesthetic method for primary reconstruction. As a valid tool for correcting postoperative contour deformities of the breast, it has also been described as a salvage flap for failed prior reconstructions (6–9). Partial mastectomy with mini latissimus dorsi flap (MLDF), as the novel improved method of mastectomy with LDF, fills the tumor cavity by using autologous flaps in selected patients and provides a cosmetically successful breast reconstruction.

With innovations in recent years, implant-based reconstruction becomes the most common method of postmastectomy breast reconstruction in use today (10). Especially with the advent of biology matrix or acellular dermal matrix (ADM), it has prompted some surgeons to consider direct-to-implant (DTI) breast reconstruction as a better alternative to tissue expander/implant-based techniques or autologous tissue reconstructions (11, 12). Previous studies showed that LDF breast reconstruction and other autologous tissue reconstructions yielded superior aesthetic results with fewer risks (9, 13). However, biological matrix-assisted DTI breast reconstruction can also provide good psychosocial benefits without the need to perform further surgery, which is suitable for China’s large population base (14, 15).

With the continuous advancement of surgical techniques, surgeons pay more attention to understanding the patient-reported outcomes (PROs) and patient satisfaction following breast reconstruction (16). More and more surgeons in China have noticed that QOL, body image, and psychosocial well-being are critically important to women after mastectomy. The BREAST-Q, which was introduced in 2009, has been widely used to evaluate PROs after breast reconstruction and can detect small clinically meaningful differences between individual patients and groups (17, 18). In this study, we use the more advanced patient-reported outcome measures (PROMs) of BREAST-Q version 2.0 to assess health outcomes from the patients’ perspective.

To our knowledge, previous studies more often compare the total autogenous reconstruction and DTI reconstruction and few studies directly compare the biological matrix-assisted DTI reconstruction and LDF reconstruction in detail. Besides, we distinguish the MLDF reconstruction from the LDF reconstruction and do a comprehensive comparison, which is lacking in previous studies. Furthermore, PRO analyses are missing from most previous studies. This study aims to assess PROs and complications between the immediate autologous latissimus dorsi flap (LDF) breast reconstruction and biological matrix-assisted DTI breast reconstruction. The second purpose of the study is to compare and analyze the PROs and complications between the mastectomy with LDF breast reconstruction and partial mastectomy with MLDF breast reconstruction.

## 2 Methods

### 2.1 Study Population

This research was a retrospective cohort study based on Chinese population characteristics to compare long-term outcomes between traditional LDF breast reconstruction and currently popular biological matrix-assisted DTI breast reconstruction. Patients in our institution were recruited from August 2010 to April 2019. Eligible patients included all women aged 18 years old and older presenting for first-time breast reconstruction with LDF or one-stage implant for cancer treatment at the National Cancer Center of China. Patients who had contraindications with porcine were excluded from the SIS matrix-assisted DTI breast reconstruction group. The postoperative follow-up of patients was at least 1 year. Patients were then divided into three groups: those who underwent LDF reconstruction, those who underwent MLDF reconstruction, and patients who underwent SIS matrix-assisted DTI breast reconstruction. Written informed consent forms were obtained from all patients. This study conformed with the Declaration of Helsinki and was approved by the Center of Clinical Research, Cancer Hospital, Chinese Academy of Medical Sciences and Peking Union Medical College.

### 2.2 Questionnaire Collection

The BREAST-Q version 2.0 questionnaire was a validated instrument developed at Memorial Sloan Kettering Cancer Center and the University of British Columbia measuring patients’ satisfaction and health-related QOL following different breast surgical procedures (17, 19).

The postoperative BREAST-Q version 2.0 PROM included the following: 1) the health-related QOL domain (psychosocial well-being, physical well-being: chest, and sexual well-being); 2) the satisfaction domain (satisfaction with breasts); 3) the experience domain (satisfaction with information, satisfaction with surgeon). Patients who underwent DTI breast reconstruction were incorporated with a special scale: satisfaction with implant. Comparably, patients who underwent LDF breast reconstruction were incorporated with other scales: satisfaction with back, physical well-being: back and shoulder.

Using the corresponding scoring table, values for BREAST-Q version 2.0 subscales were converted to the equivalent Rasch-transformed scores that ranged from 0 to 100. Except for physical well-being: chest, satisfaction with back and physical well-being: back and shoulder, higher scores reflected a superior patient satisfaction or better QOL. Surveys were completed online with BREAST-Q version 2.0 questionnaires through mobile phones.

### 2.3 Sociodemographic and Clinicopathological Materials

A retrospective electronic medical record review was performed for a consecutive series of patients undergoing immediate breast reconstruction using SIS matrix-assisted DTI reconstruction or LDF or MLDF reconstruction. Proportions of estrogen receptor (ER)-positive, progesterone receptor (PR)-positive, human epidermal growth factor receptor 2 (HER2)-positive, Ki67>20% of patients were matched between groups with no statistical differences. The following demographic information was compiled: age, body mass index (BMI), laterality (left or right), tumor size of the postoperative pathology, tumor location, lymph node management (sentinel lymph node or axillary lymph node dissection), surgical type [nipple-sparing mastectomy/skin-sparing mastectomy (NSM/SSM)], duration of surgery, time to drain removal, neoadjuvant/adjuvant chemotherapy, radiation therapy, hormone therapy, targeted therapy, smoking status (current or former), diabetes, and the length of follow-up.

### 2.4 Complications

Postoperative complications were also evaluated, and all the complications were divided into major complications and minor complications. A complication is defined as an adverse, postoperative, surgery-related event requiring additional treatment. Major complications are designated as those requiring rehospitalization or reoperation. And minor complications are those that can be treated in dressing rooms or treated with drugs. Complications included seroma, infections, implant loss, tumor recurrence/metastasis, dehiscence, nipple–areola complex (NAC) necrosis, and chronic pain.

### 2.5 Indications and Surgical Technique

**Figure 1:**
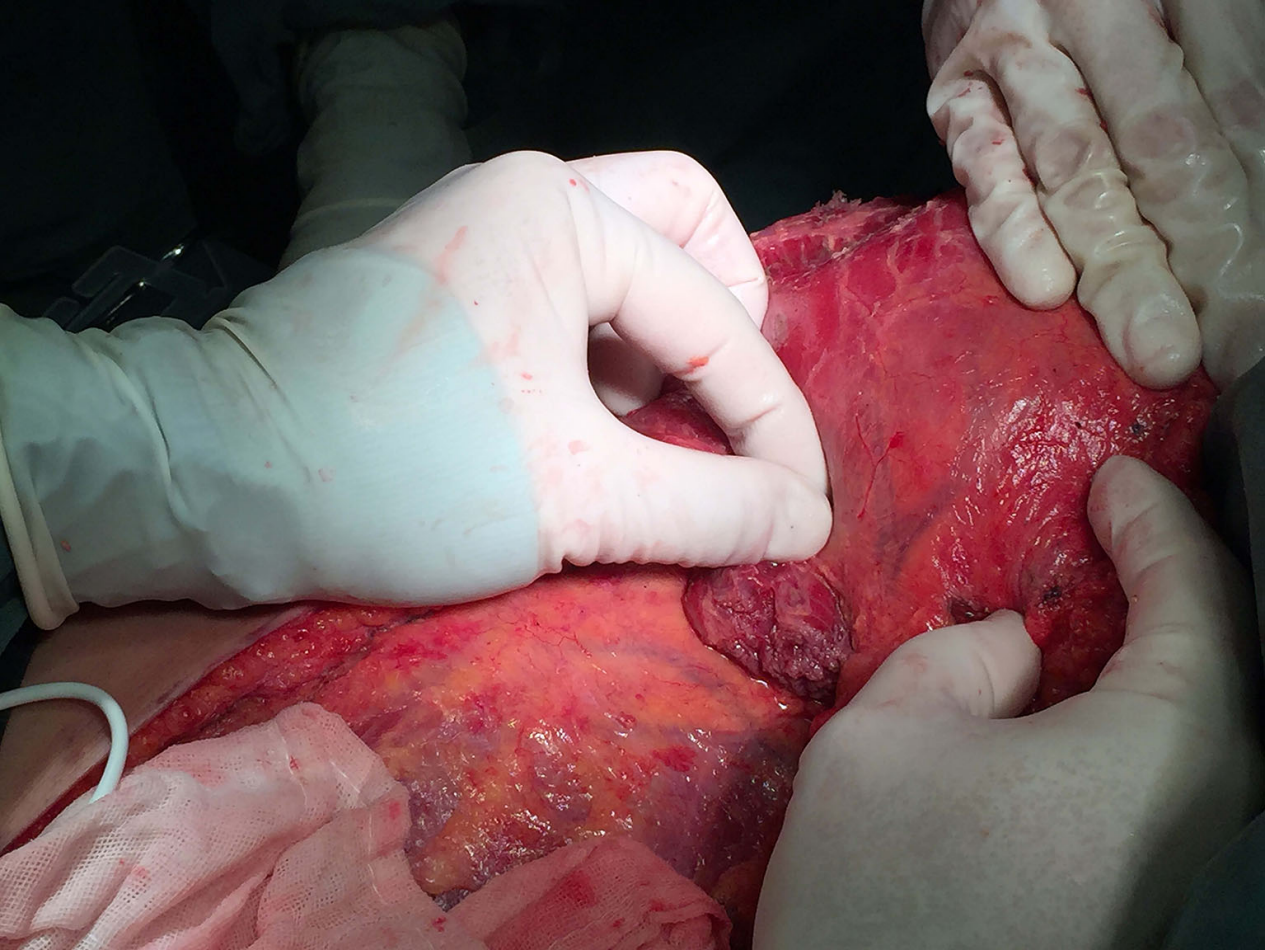
A 42-year-old woman who was diagnosed with invasive carcinoma of the breast and axillary lymph node metastasis. The surgeon was protecting the vessels to separate the latissimus dorsi muscle during latissimus dorsi flap breast reconstruction.

These three different surgical techniques are all immediate one-step procedures. Before surgery, all patients are diagnosed with breast cancer with pathology. Operation methods are selected by the surgeon according to the patient’s condition with comprehensive evaluation before surgery. LDF or MLDF breast reconstruction is for patients with axillary lymph node metastasis who have a high possibility of receiving postoperative radiation. Partial mastectomy with MLDF breast reconstruction is given for patients who had a large tumor with a strong desire to conserve the breast. At the same time, the breast of the patients should not be too small and the tumor is better located at the outer quadrant or upper quadrant. Patients who receive mastectomy with LDF breast reconstruction have high tumor/breast ratios but a small breast. The goal of the autologous LDF breast reconstruction is to minimize the magnitude of donor site defect and donor site complications, while maximizing the soft tissue coverage provided by the flap. **Figure 1** showed the intraoperative scene when the surgeon was separating the latissimus dorsi muscle in the LDF breast reconstruction. Nearly all patients who undergo MLDF breast reconstruction are marked with titanium clips that aim for the postoperative radiation during oncoplastic partial mastectomy.

For the patients who undergo mastectomy with DTI breast reconstruction, the inferior and outer origins of the pectoralis major muscle are released to create a subpectoral pocket. Then, a definitive implant is placed below the muscle. One type of biological matrix is used to be fixed to the chest wall to cover and support the lower and lateral areas of the implant. In this study, we chose the SIS matrix (Biodesign Surgisis, Cook Biotech), which is derived from porcine small intestine submucosa, to completely close the pocket according to the insufficiency of pectoralis major muscle.

During the perioperative period, all rules for hygienic prosthetic surgery are followed meticulously to reduce the likelihood of bacterial contamination. Patients with implant reconstruction will receive one prophylactic dose of an antibiotic 30 min before surgical incision and two doses in the 24 h after surgery. The drains remain in place until the output is less than 20 ml within 24 h for 3 consecutive days.

### 2.6 Statistical Analysis

Patient characteristics and complications were compared between the three procedure types using Student’s t-test for continuous variables and chi-square test for categorical variables. For all analyses, the patient was the analytic unit.

For PROs, mean scores of postoperative BREAST-Q Version 2.0 were summarized by the two procedure types. To compare the PROs between the two procedure types, Student’s t-test was used for each PROM.

All statistical analyses were performed by IBM SPSS (version 25), and the level of significance used for all analyses was two-tailed and set at p < 0.05.

## 3 Results

During the study period, a total of 135 patients met the inclusion criteria. Twenty-nine patients (21.5%) underwent LDF breast reconstruction, 27 patients (20%) underwent MLDF breast reconstruction, and 79 patients (58.5%) underwent SIS matrix-assisted DTI breast reconstruction. For patients who underwent LDF breast reconstruction, 55.2% (n = 16) responded to the BREAST-Q version 2.0 survey, and 70.4% of patients (n = 19) undergoing MLDF breast reconstruction responded to the survey, whereas 86.1% of patients (n = 69) with SIS matrix-assisted DTI breast reconstruction were responders.

### 3.1 LDF Group vs. MLDF Group

#### 3.1.1 Sociodemographic Results and Medical History

When comparing patients who underwent LDF breast reconstruction and MLDF breast reconstruction, all patients in the two groups underwent unilateral reconstructions, and patients were similar in average age, tumor size of postoperative pathology, tumor locations, lymph node management, time to drain removal, smoking status, and diabetes mellitus (**Table 1**). However, patients who underwent MLDF breast reconstruction had a shorter duration of surgery with 3.28 ± 0.69 h compared with 3.77 ± 0.63 h in the LDF group (p = 0.008). Although there was no statistical significance, patients who underwent MLDF breast reconstruction had a slightly larger body mass index at 25.51 ± 6.88 kg/m^2^ and slightly larger tumor size of 3.25 ± 1.60 cm compared with the body mass index at 22.76 ± 2.68 kg/m^2^ (p = 0.060) and tumor size of 2.76 ± 2.03 cm (p = 0.320) in the LDF breast reconstruction group.

**Table 1:** Baseline characteristics of the included patients who received LDF or MLDF breast reconstruction.

Characteristics	LDF (n = 29)	MLDF (n = 27)	p^*^
Mean age, years (SD)	40.34 (9.00)	38.63 (7.75)	0.450
Mean BMI, kg/m^2^ (SD)	22.76 (2.68)	25.51 (6.88)	0.060
Unilateral reconstruction, n (%)			0.100
Right	16 (55.2)	9 (33.3)	
Left	13 (44.8)	18 (66.7)	
Tumor size (postoperative pathology), cm (SD)	2.76 (2.03)	3.25 (1.60)	0.320
Tumor location, n (%)			0.508
Upper outer quadrant	21 (72.4)	19 (70.4)	
Lower outer quadrant	1 (3.4)	2 (7.4)	
Lower inner quadrant	4 (13.8)	1 (3.7)	
Upper inner quadrant	3 (10.3)	4 (14.8)	
Others	0	1 (3.7)	
Lymph node management, n (%)			
Axillary resection^#^	26 (89.7)	24 (88.9)	1.000
Sentinel node	6 (20.7)	6 (22.2)	0.889
Surgical type, n (%)			<0.001
NSM	12 (41.4)	25 (92.6)	
SSM	17 (58.6)	2 (7.4)	
Duration of surgery, hours (SD)	3.77 (0.63)	3.28 (0.69)	0.008
Time to drain removal^#^, days (SD)	27.87 (12.41)	29.14 (11.26)	0.691
Treatment, n (%)			
Neoadjuvant chemotherapy	5 (17.2)	7 (25.9)	0.429
Adjuvant chemotherapy	22 (75.9)	24 (88.9)	0.356
Radiotherapy status	11 (37.9)	24 (88.9)	<0.001
Hormone therapy	18 (62.1)	19 (70.4)	0.512
Targeted therapy^#^	4 (13.8)	9 (33.3)	0.157
Smoking status, n (%)			
Former	2 (6.9)	2 (7.4)	1.000
Current	0	0	
Diabetes mellitus, n (%)	2 (6.9)	1 (3.7)	1.000
Mean duration of follow-up at time of BREAST-Q, months (SD)	75.33 (28.72)	59.59 (23.03)	0.096

BMI, body mass index; LDF, latissimus dorsi flap; MLDF, mini latissimus dorsi flap; NSM, nipple-sparing mastectomy; SD, standard deviation; SSM, skin-sparing mastectomy.

^#^Axillary resection means axillary lymph node dissection (ALND). Time to drain removal is the extraction time of the last drainage tube after reconstruction. Targeted therapy means anti-HER2 therapy.

*Statistically significant difference (p < 0.05).

Continuous variables were analyzed using Student’s t-test; categorical variables were analyzed using chi-square test.

NSM was more commonly performed for MLDF patients (p < 0.001), and radiation therapy was more common in the MLDF breast reconstruction group compared with patients in the LDF group (p < 0.001). Other treatments were not significantly different between the two groups.

#### 3.1.2 BREAST-Q Version 2.0 Scores

**Figure 2:**
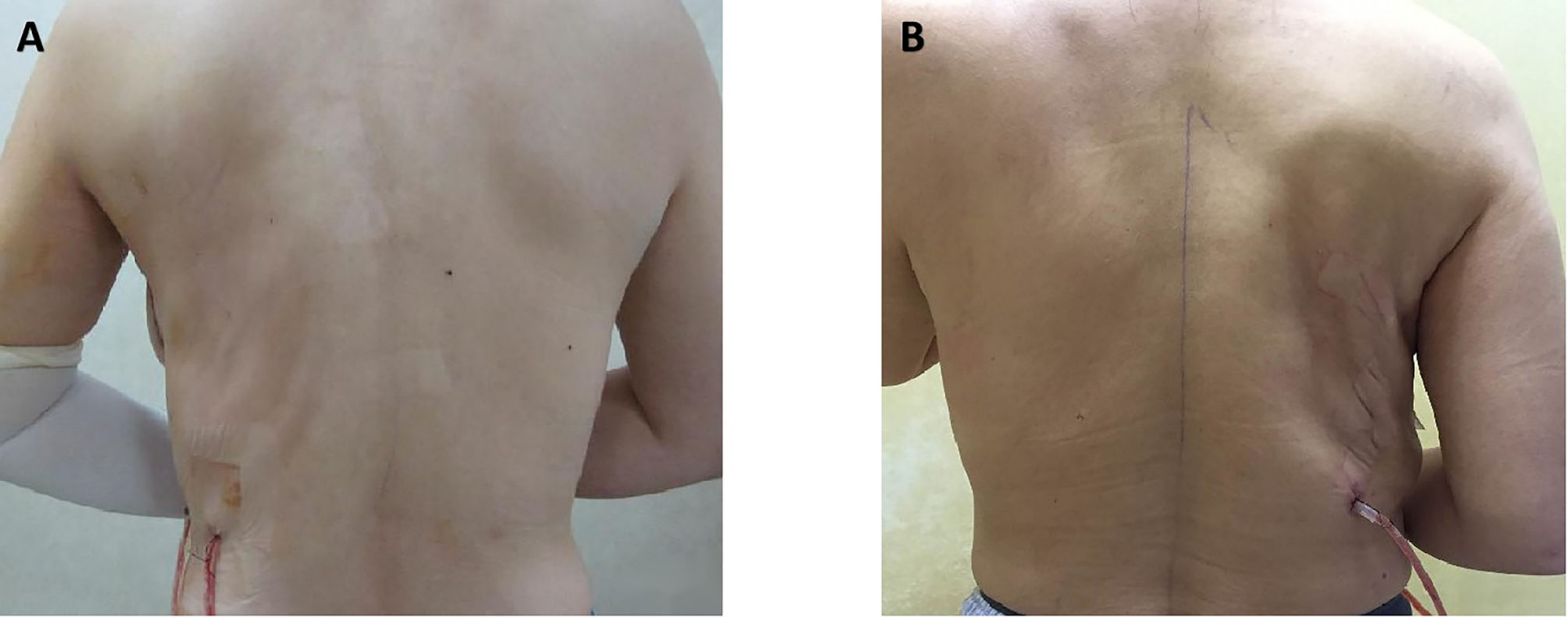
Comparison of the back shapes after breast reconstruction at 1 week. The back photograph of a patient who underwent partial mastectomy with mini latissimus dorsi flap breast reconstruction **(A)**. The back photograph of a patient who underwent mastectomy with latissimus dorsi flap breast reconstruction **(B)**.

Means for postoperative PRO scores from the BREAST-Q version 2.0 were summarized in **Table 2**. This study especially compared the subscales of satisfaction with back and Physical Well-Being: Back and Shoulder between the two groups. Even though patients in the MLDF group yielded better outcomes than patients in the LDF group (lower scores reflected a superior patient satisfaction or better QOL in these two subscales), no statistical significance was found between the two traditional LDF breast reconstruction groups in the health-related QOL domain, the satisfaction domain, and the experience domain. **Figure 2** showed the comparison photographs of the back shapes after MLDF and LDF breast reconstruction.

**Table 2:** Postoperative scales of BREAST-Q Version 2.0 between LDF and MLDF breast reconstruction.

Items	LDF reconstruction		MLDF reconstruction		p^*^
	Data available, n (%) of N	Mean (SD)	Data available, n (%) of N	Mean (SD)	
Psychosocial well-being	16 (55.2%) of 29	87.69 (18.94)	19 (70.4%) of 27	81.47 (15.69)	0.296
Satisfaction with breast	16 (55.2%) of 29	56.38 (16.69)	19 (70.4%) of 27	66.00 (14.35)	0.076
Satisfaction with back	16 (55.2%) of 29	52.50 (29.44)	19 (70.4%) of 27	41.79 (25.11)	0.254
Physical Well-Being: Back and Shoulder	16 (55.2%) of 29	40.63 (25.00)	19 (70.4%) of 27	38.05 (18.94)	0.731
Physical well-being: chest	16 (55.2%) of 29	39.31 (24.52)	19 (70.4%) of 27	33.21 (16.11)	0.384
Sexual well-being	10 (34.5) of 29	70.43 (24.97)	12 (44.4) of 27	69.10 (25.74)	0.917
Satisfaction with information	16 (55.2%) of 29	66.19 (18.86)	19 (70.4%) of 27	72.05 (19.85)	0.380
Satisfaction with surgeon	16 (55.2%) of 29	85.38 (27.94)	19 (70.4%) of 27	79.53 (17.54)	0.456

LDF, latissimus dorsi flap; MLDF, mini latissimus dorsi flap; SD, standard deviation.

*Statistically significant difference (p < 0.05).

Student’s t-test was used for each patient-reported outcome measure.

#### 3.1.3 Complications

When comparing complications in the LDF breast reconstruction group to the MLDF breast reconstruction group, patients had similar incidences of major complications at 6.9% (n = 2) vs. 11.1% (n = 3) (p = 0.664). During the follow-up, we found that nearly all the major complications were due to tumor metastasis. Meanwhile, patients in the LDF breast reconstruction group had similar incidences of minor complications compared with the MLDF breast reconstruction group (**Table 3**). And the highest minor complication rate in the two groups of patients was both seroma.

**Table 3:** Univariate analysis results of the complications between LDF breast reconstruction and MLDF breast reconstruction.

Complications	LDF (n = 29)	MLDF (n = 27)	p^*^
Major complications, n (%)	2 (6.9)	3 (11.1)	0.664
Minor complications, n (%)	4 (13.8)	2 (7.4)	0.671
Seroma, n (%)	3 (10.3)	3 (11.1)	1.000
Infections, n (%)	0	0	
Recurrence/metastasis, n (%)	2 (6.9)	2 (7.4)	1.000
NAC necrosis, n (%)	0	0	
Chronic pain, n (%)	1 (3.4)	0	1.000

LDF, latissimus dorsi flap; MLDF, mini latissimus dorsi flap; NAC, nipple–areola complex.

*Statistically significant difference (p < 0.05).

Categorical variables were analyzed using chi-square test.

### 3.2 The Whole Autologous LDF Reconstruction Group vs. DTI Reconstruction Group

According to the baseline characteristics, PROs, and complication rates, we found that there were no obvious statistical significances between the LDF breast reconstruction group and the MLDF breast reconstruction group. Then, we decided to compare the whole autologous LDF reconstruction group (LDF reconstruction + MLDF reconstruction) with the SIS matrix-assisted DTI breast reconstruction group.

#### 3.2.1 Sociodemographic Results and Medical History

**Figure 3:**
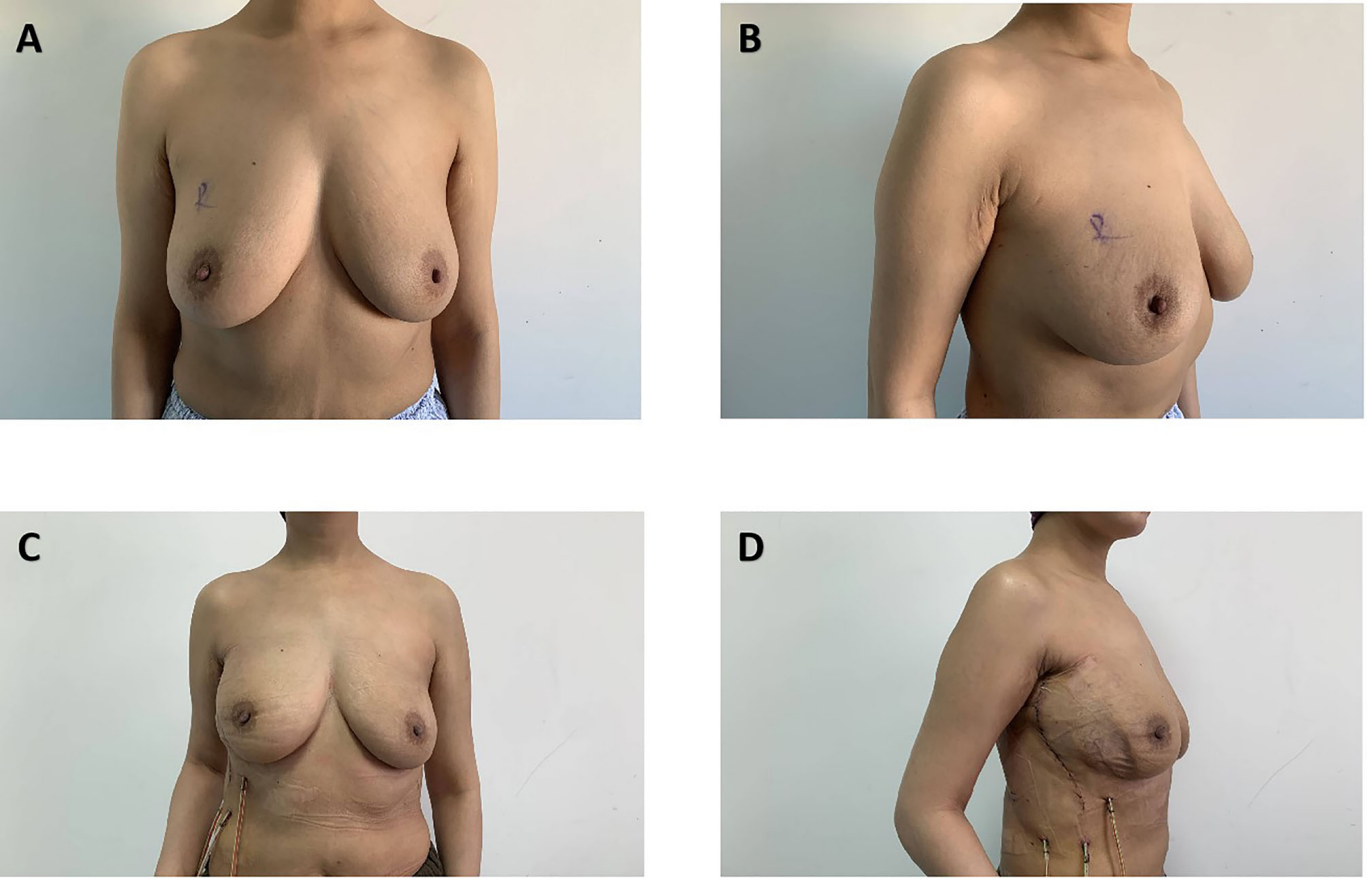
Unilateral right nipple-sparing mastectomy with mini latissimus dorsi flap breast reconstruction. A 44-year-old woman who was diagnosed with invasive carcinoma of the right breast and axillary lymph node metastasis before surgery **(A, B)**. Photos at 7 days postoperatively **(C, D)**.

**Figure 4:**
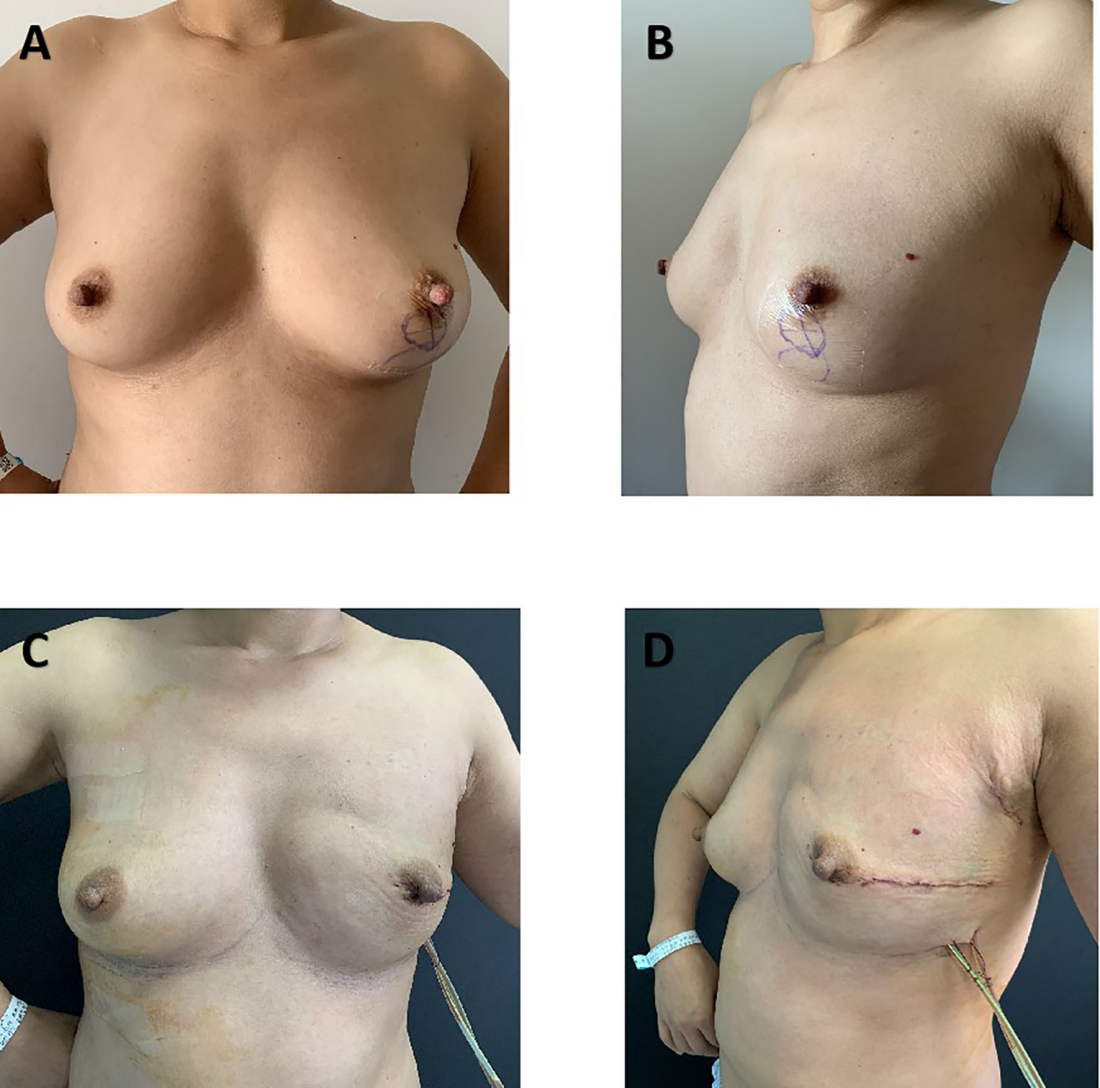
Unilateral left nipple-sparing mastectomy and immediate direct-to-implant breast reconstruction with SIS matrix. A 40-year-old woman who was diagnosed with invasive carcinoma and was treated with neoadjuvant chemotherapy before surgery **(A, B)**. Photos at 7 days postoperatively **(C, D)**.

Of the overall groups, 79 patients underwent the SIS matrix-assisted DTI breast reconstruction and a total of 56 patients underwent the autologous LDF reconstruction. These two groups differed in several variables (**Table 4**). Patients who underwent the SIS matrix-assisted DTI reconstruction had a slightly smaller body mass index at 21.73 ± 2.20 kg/m^2^ compared with the whole autologous LDF reconstruction at 24.08 ± 5.28 kg/m^2^ (p = 0.002). The result of postoperative pathology showed that the tumor size in the whole autologous LDF reconstruction group was larger than the SIS matrix-assisted DTI reconstruction group (3.00 ± 1.82 cm vs. 2.35 ± 1.24 cm; p = 0.022). Expectedly, axillary resection was more commonly performed for autologous LDF breast reconstruction (p < 0.001). The duration of surgery (p < 0.001) and the time to drain removal (p < 0.001) in the whole autologous LDF reconstruction group were obviously longer compared with those in the SIS matrix-assisted DTI breast reconstruction. For treatment, adjuvant chemotherapy (0.003) and axillary dissection (p < 0.001) were more common in the whole autologous LDF reconstruction group. The distributions of age, laterality, tumor location, surgical type (NSM/SSM), other treatments, smoking status, and diabetes mellitus were not significantly different between the two groups. **Figure 3** showed the comparative photographs before and after MLDF breast reconstruction, and **Figure 4** showed the comparative photographs before and after SIS matrix-assisted DTI reconstruction.

**Table 4:** Baseline characteristics of the included patients who received DTI or autologous LDF breast reconstruction.

Characteristics	SIS matrix-assisted DTI (n = 79)	The whole autologous LDF reconstruction (n = 56)	p^*^
Mean age, years (SD)	41.14 (7.03)	39.52 (8.39)	0.226
Mean BMI, kg/m^2^ (SD)	21.73 (2.20)	24.08 (5.28)	0.002
Unilateral reconstruction, n (%)			0.801
Right	37 (46.8)	25 (44.6)	
Left	42 (53.2)	31 (55.4)	
Tumor size (postoperative pathology), cm (SD)	2.35 (1.24)	3.00 (1.82)	0.022
Tumor location, n (%)			0.189
Upper outer quadrant	43 (54.4)	40 (71.4)	
Lower outer quadrant	14 (17.7)	3 (5.4)	
Lower inner quadrant	7 (8.9)	5 (8.9)	
Upper inner quadrant	12 (15.2)	7 (12.5)	
Others	3 (3.8)	1 (1.8)	
Lymph node management, n (%)			
Axillary resection^#^	32 (40.5)	50 (89.3)	<0.001
Sentinel node	52 (65.8)	12 (21.4)	
Surgical type, n (%)			0.227
NSM	59 (74.7)	37 (66.1)	
SSM	20 (25.3)	19 (33.9)	
Duration of surgery, h (SD)	2.04 (0.43)	3.53 (0.70)	<0.001
Time to drain removal^#^, days (SD)	22.03 (8.76)	28.48 (11.78)	<0.001
Treatment, n (%)			
Neoadjuvant chemotherapy	9 (11.4)	12 (21.4)	0.113
Adjuvant chemotherapy	46 (58.2)	46 (82.1)	0.003
Radiotherapy status	16 (20.3)	35 (62.5)	<0.001
Hormone therapy	52 (65.8)	37 (66.1)	0.976
Targeted therapy^#^	20 (25.3)	13 (23.2)	0.779
Smoking status, n (%)			
Former	4 (5.1)	2 (3.6)	1.000
Current	0	0	
Diabetes mellitus, n (%)	0	1 (1.8)	0.415
Mean duration of follow-up at time of BREAST-Q, months (SD)	17.55 (4.83)	66.97 (26.64)	<0.001

BMI, body mass index; DTI, direct-to-implant; LDF, latissimus dorsi flap; NSM, nipple-sparing mastectomy; SD, standard deviation; SSM, skin-sparing mastectomy.

^#^Axillary resection means axillary lymph node dissection (ALND). Time to drain removal is the extraction time of the last drainage tube after reconstruction. Targeted therapy means anti-HER2 therapy.

*Statistically significant difference (p < 0.05).

Continuous variables were analyzed using Student’s t-test; categorical variables were analyzed using chi-square test.

#### 3.2.2 BREAST-Q Version 2.0 Scores

The BREAST-Q version 2.0 scores of the three domains for the SIS matrix-assisted DTI breast reconstruction group vs. the whole autologous LDF reconstruction group were compared (**Table 5**). The return rate of the BREAST-Q in the DTI reconstruction group was 86.1%. And the return rate of the questionnaire in the whole autologous LDF reconstruction group was 64.3%. Patients in the whole autologous LDF reconstruction group had greater psychosocial well-being after surgery, showing a mean score of 84.31 ± 17.28, compared with the DTI breast reconstruction, with a mean score of 73.52 ± 19.96 (p = 0.008). Patients in the whole autologous LDF reconstruction group also expressed higher sexual well-being at 69.65 ± 24.64 vs. 50.95 ± 26.47 (p = 0.016). No statistical significance was observed regarding the other subscales between the two groups.

**Table 5:** Postoperative scales of BREAST-Q Version 2.0 between DTI and autologous LDF breast reconstruction.

Items	SIS matrix-assisted DTI		The whole autologous LDF reconstruction		p^*^
	Data available, n (%) of N	Mean (SD)	Data available, n (%) of N	Mean (SD)	
Psychosocial well-being	68 (86.1%) of 79	73.52 (19.96)	36 (64.3%) of 56	84.31 (17.28)	0.008
Satisfaction with breast	68 (86.1%) of 79	60.27 (17.71)	36 (64.3%) of 56	61.60 (15.99)	0.710
Physical well-being: chest	68 (86.1%) of 79	39.60 (17.39)	36 (64.3%) of 56	36.00 (20.30)	0.352
Sexual well-being	40 (50.6%) of 79	50.95 (26.47)	22 (39.3%) of 56	69.65 (24.64)	0.016
Satisfaction with information	68 (86.1%) of 79	66.79 (18.15)	36 (64.3%) of 56	69.37 (19.35)	0.507
Satisfaction with surgeon	68 (86.1%) of 79	85.49 (21.38)	36 (64.3%) of 56	82.20 (22.71)	0.472

DTI, direct-to-implant; LDF, latissimus dorsi flap; SD, standard deviation.

SIS matrix (Biodesign Surgisis, Cook Biotech).

*Statistically significant difference (p < 0.05).

Student’s t-test was used for each patient-reported outcome measure.

#### 3.2.3 Complications

Bivariate analyses on postoperative complications are summarized in **Table 6**. When comparing complications in the whole autologous LDF reconstruction group to the SIS matrix-assisted DTI group, patients had similar incidences of major complications at 8.9% (n = 5) vs. 6.3% (n = 5) (p = 0.570) and similar incidences of minor complications at 10.7% (n = 6) vs. 11.4% (n = 9) (p = 0.902). However, patients who underwent autologous LDF breast reconstruction had higher incidences of tumor metastasis at 7.1% (n = 4) vs. 0% (n = 0) (p = 0.028). Patients who underwent the SIS matrix-assisted DTI breast reconstruction had higher incidences of infectious complications at 5.1% (n = 4) vs. 0% (n = 0) (p = 0.141), but this observation failed to reach statistical significance. By contrast, there were 4 patients in the SIS matrix-assisted DTI breast reconstruction group losing the implants to have secondary revision operations [5.1% (n = 4)].

**Table 6:** Univariate analysis results of the complications between DTI and autologous LDF breast reconstruction.

Complications	SIS matrix-assisted DTI (n = 79)	The whole autologous LDF reconstruction (n = 56)	p^*^
Major complications, n (%)	5 (6.3)	5 (8.9)	0.570
Minor complications, n (%)	9 (11.4)	6 (10.7)	0.902
Seroma, n (%)	6 (7.6)	6 (10.7)	0.530
Infection, n (%)	4 (5.1)	0	0.141
Implant loss, n (%)	5 (6.3)	0	0.076
Metastasis, n (%)	0 (0)	4 (7.1)	0.028
Dehiscence, n (%)	2 (2.5)	0	0.511
NAC necrosis, n (%)	2 (2.5)	0	0.511
Chronic pain, n (%)	2 (2.5)	1 (1.8)	1.000

DTI, direct-to-implant; LDF, latissimus dorsi flap; NAC, nipple–areola complex.

*Statistically significant difference (p < 0.05).

Categorical variables were analyzed using chi-square test.

## 4 Discussion

Women are faced with a complex, multilayered reconstruction decision as they choose after breast cancer surgery. The significant demand for breast reconstruction brought about the development of a myriad of acceptable procedures (20, 21). Implant-based reconstruction, as the most popular reconstruction method today, includes tissue expander/implant reconstruction and DTI reconstruction. Compared with the tissue expander/implant reconstruction, the biological matrix-assisted reconstruction is a single-stage approach that avoids further surgery and saves the cost for patients. Although implant-based techniques remain the most common, autologous reconstructions constitute a stable proportion worldwide (22). For breast oncology surgeons, choosing the right operation usually involves a careful weighing of the potential benefits against the risks of the various procedure types. For example, previous studies showed that radiation therapy was considered a relative contraindication to implant-based reconstruction (6). These comprehensive complication data affect surgeons and patients to make the optimal choice, which causes the high proportion of patients who were treated with radiotherapy and adjuvant chemotherapy in the whole autologous LDF reconstruction group in our study. This study provides a comprehensive analysis and comparison in terms of complications and PROs between biological matrix-assisted DTI reconstruction and autologous LDF reconstruction, which further standardized various surgical indications and made the reconstruction surgery process clearer.

The latissimus dorsi muscle skin flap that was used to close the mastectomy defect was first reported in 1906, but the technique did not gain popularity in breast reconstruction until the 1970s. And it started to be used also in partial mastectomies after the 1990s (23, 24). MLDF reconstruction provides a cosmetically successful breast reconstruction for patients with a large tumor but a large breast volume. The possibility to fill the tumor cavity by using MLDF in selected patients enables this intervention to be a serious alternative to subcutaneous mastectomy with LDF (25), and few studies compare these two different LDF breast reconstructions. Thus, we directly compare PROs and complications of LDF reconstruction and MLDF reconstruction in our study. The numbers of patients in the two groups are approximate (n = 29 vs. n = 27). But as a kind of innovative technology of autologous LDF breast reconstruction in our cancer center, the time of follow-up of the MLDF reconstruction group is shorter than that of the LDF reconstruction group. However, outcomes show no statistical difference in major and minor complications between the two groups. And PROs also demonstrate no significant statistical difference despite that more patients in the MLDF reconstruction group save the NAC and had better back shapes, which might yield a positive psychologic effect on patients’ mood. But it is worth noting that the duration of surgery in the MLDF reconstruction group was half an hour shorter than that in the LDF reconstruction group (3.28 ± 0.69 h vs. 3.77 ± 0.63 h, p = 0.008).

Subcutaneous mastectomy with implant usually causes asymmetries and required additional cosmetic approaches (25). However, with the introduction of the biological matrix in the field of breast reconstruction in 2005, the use of biological matrix or ADM makes DTI breast reconstruction feasible and lead to improved aesthetic results by creating a more natural-looking breast (26, 27).

Due to the similar outcomes between LDF reconstruction group and MLDF reconstruction group, we compare the PROs and complications of SIS matrix-assisted DTI reconstruction to the whole autologous LDF reconstruction group. Biological matrix-assisted DTI reconstruction is a single-stage approach that can be performed immediately after mastectomy avoiding further surgery (25, 28). Patients who underwent SIS matrix-assisted DTI reconstruction have a lower average body mass index compared with autologous LDF reconstruction (21.73 ± 2.20 vs. 24.08 ± 5.28, p = 0.002) and smaller tumor size (2.53 ± 1.24 vs. 3.00 ± 1.82, p = 0.022), which is measured by postoperative pathology. This is not surprising, as the obtainable overall flap volume in low–body mass index patients tends to be less than that in higher body mass index patients. And thin women with lower body mass index are more in line with Chinese women, making DTI reconstruction highly coveted by many surgeons. Compared with autologous LDF reconstruction, the duration of surgery in SIS matrix-assisted DTI reconstruction is obviously shorter (2.04 ± 0.43 vs. 3.53 ± 0.7 h, p < 0.001), and the time to drain removal is also much shorter (22.03 ± 8.76 vs. 28.48 ± 11.78 days, p < 0.001), which has significant statistical significance. These advantages of biological matrix-assisted DTI reconstruction relieve the heavy burden of surgeons, making more and more breast oncology surgeons in China choose the novel method of breast reconstruction for women with low–normal body mass index. However, is the biological matrix-assisted DTI reconstruction really better than the traditional autologous LDF reconstruction?

Breast reconstruction aims to restore physical appearance and well-being, so we choose the BREAST-Q version 2.0 to judge the value of the reconstruction by patients. But due to the longer time of follow-up of the whole autologous LDF reconstruction group, the completion rate of the BREAST-Q survey is a little lower than that of the SIS matrix-assisted DTI reconstruction group (64.3% vs. 86.1%). From this survey, we find that patients in the whole autologous LDF reconstruction group had better psychosocial well-being, showing a mean score of 84.31 ± 17.28 compared with the SIS matrix-assisted DTI reconstruction, with a mean score of 73.52 ± 19.96 (p = 0.005). This finding gets similar outcomes with previous studies, confirming an overall higher score of psychosocial well-being when comparing autologous with implant-based reconstructions (22, 29). Besides, patients who underwent autologous LDF reconstruction also expressed higher sexual well-being at 69.65 ± 24.64 vs. 50.95 ± 26.47 (p = 0.016). And patients with MLDF reconstruction also show better sexual enjoyment compared to patients with DTI reconstruction in previous studies (25). Thus, compared with the SIS matrix-assisted DTI reconstruction, autologous LDF reconstruction produces superior PROs.

Patients who underwent NSM/SSM with implant-based breast reconstruction were faced with infection, necrosis of NAC, and other complications. However, previous studies showed that biological matrix-assisted breast reconstruction reduced outpatient visits and lowered revision surgery rate and the capsular contracture rate (30–32). In our study, we analyze the minor complications that can be treated in the clinic or office setting. Outcomes show that seroma is the most common postoperative minor complication for patients with autologous LDF reconstruction, but there is no statistical significance between the two groups. We also examine the reoperative complications and the metastasis rate, which have a greater influence on patients’ lives and well-being after reconstruction. We find a total of 5 patients undergoing additional surgery with implant loss, and the main cause is infection. With a longer time of follow-up, tumor metastasis occurred in 4 patients with autologous LDF reconstruction, which has no statistical significance. Neither the SIS matrix-assisted DTI reconstruction nor the autologous LDF reconstruction has the same high complication rate as previous studies (2, 26, 33), and it does not allow the high postoperative complication rate in China with the medical environment, which is not optimistic now.

There are several limitations in our study. First, this research is a retrospective review, and the follow-up of the whole autologous LDF reconstruction group is longer than that of the SIS matrix-assisted DTI reconstruction group because the implant-based reconstruction is a newer technology in our institution. Besides, preoperative BREAST-Q investigation is not included in our study, and we cannot compare the PROs with the outcomes of preoperative baselines. Moreover, the overall number of autologous LDF reconstructions compared with SIS matrix-assisted DTI reconstructions is relatively small. This could influence the power when attempting to determine a difference between the groups. Furthermore, the incidences of adjuvant irradiation in both groups are statistically different; we recommend more patients who might have to undergo axillary resection to select autologous LDF reconstruction because previous studies reported that patients who underwent implant reconstruction with irradiation had significantly lower satisfaction with breasts compared with non-irradiated patients (34). We will continue our study with a longer period of follow-up with the reconstruction to make more contributions to the research.

Reconstruction after breast cancer surgery is increasingly becoming an essential component in the therapeutic course. With the advantage of shorter duration of surgery, the convenience to be more readily matched to the desired size, and the advent of the biological matrix to augment the subpectoral pocket, immediate implant-based reconstruction becomes the most common method of postmastectomy breast reconstruction in use today (10). However, PROs in our study show that autologous LDF reconstruction yields superior PROs than SIS matrix-assisted DTI reconstruction. And we suggest that autologous breast reconstruction is supposed to preserve a certain amount, especially for patients with higher body mass index.

## 5 Conclusion

More and more surgeons and patients are opting for biological matrix-assisted DTI reconstruction, which is the most popular reconstruction method after mastectomy. Although outcomes in our study show that the DTI reconstruction saves surgery time and is conducive to the use of health resources, better PRO benefits of the whole autologous LDF reconstruction should not be ignored. Besides, our study innovatively compares the LDF reconstruction and MLDF reconstruction, further standardizing various surgical indications and making the reconstruction surgery process clearer. Finally, our study provides surgeons and patients with new views when they do the reconstruction decision-making.

## Data Availability Statement

The data generated or analyzed during this study are included in this article, or if absent are available from the corresponding author upon reasonable request.

## Ethics Statement

This study conformed with the Declaration of Helsinki and was approved by the Center of Clinical Research, Cancer Hospital, Chinese Academy of Medical Sciences and Peking Union Medical College. Written informed consent forms were obtained from all patients.

## Author Contributions

JW: conceptualization, supervision, project administration, and funding acquisition. YF: resources, data curation, investigation, and writing—reviewing and editing. JG: formal analysis, conceptualization, methodology, validation, and visualization. PG: investigation, methodology, writing original draft preparation. PB: writing—reviewing and editing. XK: visualization and writing—reviewing and editing, and funding acquisition.

## Funding

This work was supported by the Natural Science Foundation of China (No. 81872160, No. 82072940), the China National Key R&D (or Research and Development) Program (No. 2020AAA0105000 and No. 2020AAA0105004), the Beijing Municipal Natural Science Foundation (Key Project) (No. 7191009), the Beijing Municipal Natural Science Foundation (No. 7204293), the Special Research Fund for Central Universities, Peking Union Medical College (No. 3332019053), the Beijing Hope Run Special Fund of Cancer Foundation of China (No. LC2019B03, No. LC2019L07, No. LC2020L01), the Golden Bridge Project Seed Fund of Beijing Association for Science and Technology (No. ZZ20004), the 2021 Chaoyang District Social Development Science and Technology Plan Project (Medical and Health Field) (No. CYSF2115), the Chinese Young Breast Experts Research project (No. CYBER-2021-005), the XianSheng Clinical Research Special Fund of China International Medical Foundation (No. Z-2014-06-2103), and the Beijing Xisike Clinical Oncology Research Foundation (No. Y-Young2021-0017).

## Conflict of Interest

The authors declare that the research was conducted in the absence of any commercial or financial relationships that could be construed as a potential conflict of interest.

## Publisher’s Note

All claims expressed in this article are solely those of the authors and do not necessarily represent those of their affiliated organizations, or those of the publisher, the editors and the reviewers. Any product that may be evaluated in this article, or claim that may be made by its manufacturer, is not guaranteed or endorsed by the publisher.
